# The usefulness of comprehensive genome profiling test in screening of Lynch syndrome independent of the conventional clinical screening or microsatellite instability tests

**DOI:** 10.1038/s10038-025-01345-x

**Published:** 2025-05-08

**Authors:** Mizuki Yamaguchi, Shintaro Akabane, Hiroaki Niitsu, Hikaru Nakahara, Asuka Toshida, Tetsuya Mochizuki, Takuya Yano, Yoshihiro Saeki, Hiroshi Okuda, Manabu Shimomura, Kazuhiro Sentani, Kiwamu Akagi, Hideki Ohdan, Takao Hinoi

**Affiliations:** 1 Department of Gastroenterological and Transplant Surgery, Graduate School of Biomedical and Health Science, Hiroshima University, Hiroshima, Japan; 2 Department of Clinical and Molecular Genetics, Hiroshima University Hospital, Hiroshima, Japan; 3 Department of Molecular Pathology, Graduate School of Biomedical and Health Science, Hiroshima University, Hiroshima, Japan; 4 Department of Molecular Diagnosis and Cancer Prevention, Saitama Cancer Center, Hiroshima, Japan

**Keywords:** Cancer genetics, Colorectal cancer, Cancer screening

## Abstract

Lynch syndrome (LS) is a hereditary cancer predisposition syndrome caused by germline pathogenic variants of DNA mismatch repair (MMR) genes. To diagnose LS, the microsatellite instability (MSI) test or immunohistochemistry of MMR enzymes is used as a conventional clinical screening method for all patients with colorectal and endometrial cancers. Recently, patients with advanced-stage cancers have undergone comprehensive genomic profiling (CGP), which is useful not only for the detection of molecularly targeted personalized therapies, but also for the screening of hereditary cancer syndromes by determining presumed germline pathogenic variants (PGPVs). Between January 2020 and April 2024, 1583 patients underwent CGP at our institute. PGPVs in MMR genes were detected in 19 patients. Although one patient died prior to the disclosure of the results and eight patients declined confirmatory genetic testing, the remaining ten patients underwent confirmatory genetic tests, of whom six were found to have a hereditary origin. Two additional patients were diagnosed with LS using tumor-normal paired CGP. Eventually, a total of eight patients were diagnosed with LS. Herein, we describe two patients with microsatellite-stable cancer who could not be diagnosed using conventional clinical screening or MSI testing. Furthermore, we showed that pathogenic variants of MMR genes do not always correlate with high MSI prediction scores in several cancer types in The Cancer Genome Atlas (TCGA) dataset analysis. These findings highlight the usefulness of CGP as a screening tool to identify individuals with possible LS, especially when conventional criteria and MSI/MMR testing fail.

## Introduction

In a genome, there are a number of short repeats of DNA sequences consisting of 1–6 nucleotides, which are referred to as microsatellite regions [1, 2]. During DNA replication, insertions and deletions of these repeated nucleotides in microsatellite regions occur frequently and are repaired by several mismatch repair enzymes [3]. Most microsatellite regions are found in introns, but they also exist in the exons of certain tumor suppressors and DNA repair genes [2]. Therefore, a deficiency in the mismatch repair (MMR) mechanism causes a frameshift of these coding regions in numerous loci in parallel, resulting in carcinogenesis with microsatellite instability (MSI-H) [3]. Lynch syndrome (LS) is one of the most prevalent hereditary cancer predisposition syndromes caused by germline pathogenic variants of MMR genes, including *MLH1, MSH2, MSH6, PMS2*, and, rarely*, EPCAM* [4, 5]. Owing to the presence of a germline loss-of-function variant in LS, the loss of one MMR allele is sufficient to cause MMR deficiency and carcinogenesis in multiple organs [6, 7]. Youth patients with LS have a significantly increased risk of developing colorectal and endometrial cancers. The risk of other cancers, including ovarian, gastric, small intestinal, pancreatobiliary, upper tract urothelial, brain, and skin cancers, has also increased. Additionally, evidence suggests a potential association between breast, bladder, and prostate cancers [8].

Risk stratification by diagnostic criteria based on the clinicopathological features of cancer in patients and their relatives has long been used for the screening of LS, such as the Amsterdam II criteria or the revised Bethesda guidelines (rBG) [9, 10]. Recently, universal screening by MSI testing or immunohistochemistry of MMR enzymes (MMR immunohistochemistry) for all colorectal and endometrial cancer patients has been reported to be useful for screening LS [11, 12]. Moreover, recent advances in comprehensive genomic profiling (CGP) using next generation sequencing (NGS) have enabled us to be aware of possible patients with LS who cannot be diagnosed as LS by the aforementioned strategies [13].

The CGP was approved for national health insurance coverage in Japan and has since spread rapidly across the nation [14]. CGP is useful for personalized therapy and screening for hereditary cancer syndromes [15]. Some CGP tests analyze DNA from a matched pair of tumor and normal tissues (T/N-paired CGPs), whereas others do from tumor tissues only (T-only CGPs). In contrast to T/N-paired CGPs, which report germline pathogenic variants (GPVs), only presumed germline pathogenic variants (PGPVs) were detected in T-only CGPs. Both GPVs and PGPVs are secondary findings in CGP testing and are returned to patients if desired [16, 17]. There are several recommendations for returning secondary findings from The American College of Medical Genetics (ACMG) [18], the European Society for Medical Oncology Precision Medicine Working Group (ESMO PMWG) [19], American Society of Clinical Oncology (ASCO) [20], the National Comprehensive Cancer Network (NCCN) [21], and more. Genome profiling tests enable us to be more aware of hereditary cancer syndromes than conventional clinical screening, regardless of cancer types [22–25].

In this report, we retrospectively investigated patients in whom the pathogenic variant of MMR gene was detected by CGP, and we aim to demonstrate the potential of CGP as a complementary screening strategy for Lynch syndrome, particularly in patients who would otherwise be missed by conventional clinical screening, MSI testing, or MMR immunohistochemistry.

## Methods

### Study design and participants

Between January 2020 and April 2024, 1, 583 patients underwent CGP tests for any type of solid cancer at the following institutions: Hiroshima University Hospital, Hiroshima Prefectural Hospital, NHO Kure Medical Center and Chugoku Cancer Center, Hiroshima City North Medical Center Asa Citizens Hospital, JA Onomichi General Hospital, NHO Higashihiroshima Medical Center, JA Hiroshima General Hospital, Hiroshima Red Cross Hospital and Atomic-bomb Survivors Hospital, Kagawa University Hospital, Takamatsu Red Cross Hospital, Yamaguchi University Hospital, and Shimane University Hospital. During this period, three tissue-based and two blood-based CGP tests were clinically approved and covered by the health insurance system in Japan (Supplementary Table 1), whereas several CGP tests were performed in clinical trials and private medical care. The selection of the CGP was based on the physician’s choice and patient consent. In the CGP tests, the pathogenicity of variants was determined based on annotation in CGP tests provided by companies and public databases, including ClinVar [26], OncoKB [27], InterVar [28], and VarSome (https://varsome.com); pathogenic and likely pathogenic variants in these databases were determined as pathogenic variants in this study. We also excluded single-nucleotide polymorphisms by population allele frequency in the gnomAD whole-genome sequencing [29] and jMorp/ToMMo [30] databases. GPVs and PGPVs are disclosed to patients according to a list of secondary findings to be disclosed to patients at the level of recommendation by Kosugi’s group (https://www.ncc.go.jp/jp/c_cat/jitsumushya/030/Potentially_Actionable_SF_Gene_List_Ver4.2_20231003.pdf). For MMR genes, a variant allele frequency (VAF) greater than 0.1 is considered as PGPV to be disclosed with proper genetic counseling if the patient desires. However, the final decision for disclosure was made by a germline board that was held for all of the cases of CGP testing at Hiroshima University Hospital and cooperative hospitals, considering the patients’ phenotype, family history, and VAFs. The germline board consisted of attending physicians, two certified medical geneticists, and two certified genetic counselors. For PGPVs, confirmatory genetic tests were proposed when they were disclosed to the patients. If the patients agreed to participate, germline testing was performed as part of a clinical study (Dial Study, PI: Kiwamu Akagi, Saitama Cancer Center).

In this study, we investigated the characteristics of 21 patients with either GPV or PGPV in MMR genes as follows: age at CGP testing, sex, type of CGP test, tumor mutational burden (TMB), microsatellite status, MMR immunohistochemistry, alteration and VAF of MMR genes, the presence of a personal or family history of LS-associated cancer, performance of confirmation genetic tests, and cancer type.

### Immunohistochemistry for mismatch repair enzymes

To test if there is MMR deficiency, we underwent immunohistochemistry for MLH1, PMS2, MSH2, and MLH6 by VENTANA MMR IHC Panel which includes VENTANA anti-MLH1(M1), anti-PMS2 (A16-4), anti-MSH2 (G219-1129), and anti-MSH6 (SP93) mouse monoclonal antibodies, respectively. All staining were performed by a VENTANA BenchMark ULTRA instrument with the OptiView DAB IHC Detection Kit and ancillary reagents which are approved as companion diagnostics.

### Analyzing the cancer genome atlas (TCGA), pancancer atlas dataset

We investigated whether MANTIS and MSIsensor scores, both of which are established MSI prediction scores, were affected by the presence or absence of pathogenic variants of MMR gene, including *MLH1, MSH2, MSH6*, and *PMS2* among the 30 cancer types in the TCGA Pancancer Atlas dataset. The MANTIS score ≥0.4 was defined as microsatellite instability-high (MSI-H), while MSIsensor score ≥3 and ≥10 were defined as MSI intermediate (MSI-int) and MSI-H, respectively.

## Results

### Patient characteristics

**Fig. 1:**
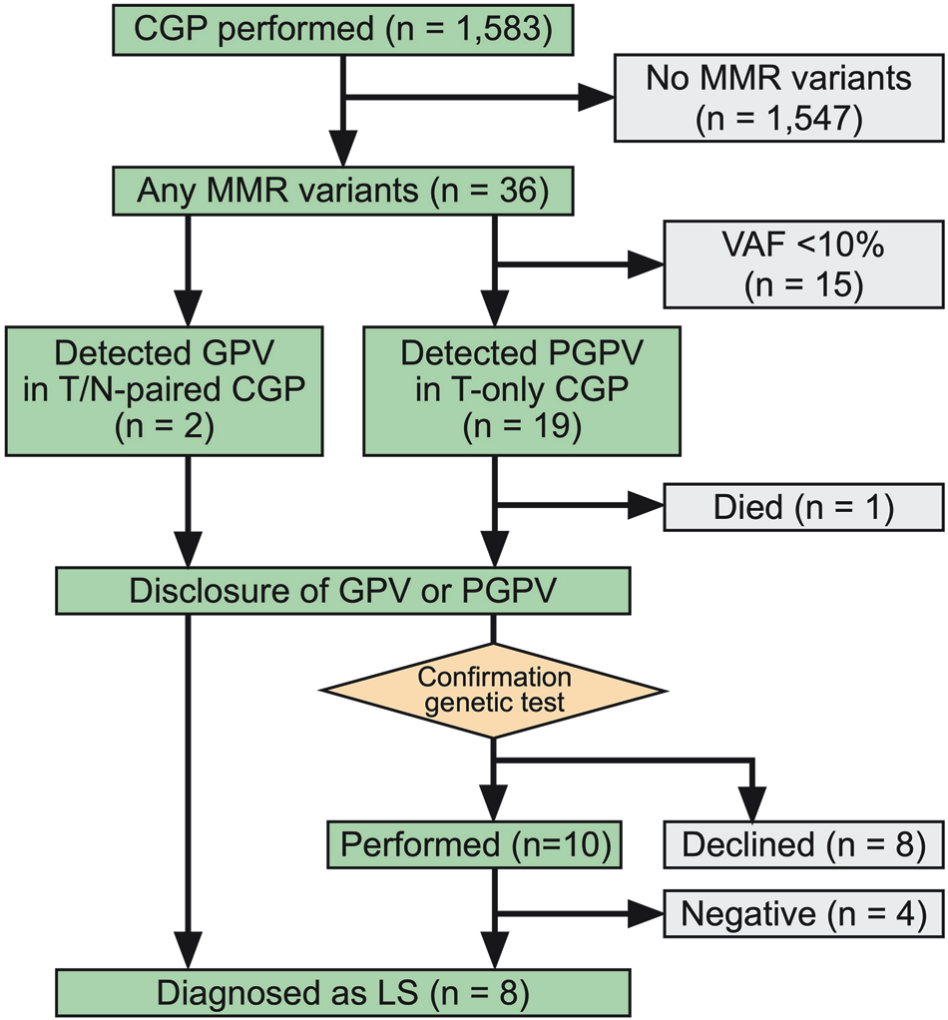
A consort diagram of the study. CGP comprehensive genomic profiling, MMR mismatch repair, VAF variant allele frequency, PGPV presumed germline pathogenic variant, GPV germline pathogenic variant, LS lynch syndrome

Among 1583 patients who underwent CGP between January 2020 and April 2024 at our institute, pathogenic variants of the MMR gene were detected in 36. Information on the 1583 cases, including the cancer type and CGP test type, is provided in Supplementary Table 2. From these patients, PGPVs were determined by T-only CGP tests in 19 patients, guided by the patients’ phenotype, family history, and a VAF greater than 0.1. The two GPVs were determined using T/N-paired CGP tests. Both PGPV and GPV were disclosed in all patients (Fig. 1 and Table 1). The median age of the 21 patients was 60 years (range, 9–81 years); 12 and 9 were male and female, respectively. MSI-H was observed in 10 patients, whereas the remaining 11 patients were microsatellite-stable (MSS). Pathogenic variants of the MMR gene were distributed as follows: *PMS2* in eight patients, *MSH6* in three patients, *MLH1* in five patients, and *MSH2* in five patients. Colorectal and uterine cancers accounted for only 33% (7 of 21) of the cases, although they are the two major LS-associated cancers. Of the 21 patients, eight were finally diagnosed with LS (Fig. 1). Only two cases of colorectal cancer were included in these patients, and there was no characteristic trend in the personal/family history of LS-associated cancer (Table 1). We included two patients who had MSS cancer but were found to have germline pathogenic variants of MMR genes as a result of CGP tests, although this was difficult to identify through conventional clinical screening.

**Table 1:** Summary of patients with GPV/PGPV of MMR gene indicated by CGP testing

Case	Sex	Age	CGP test	Tumor/ Normal	TMB	MSI	MMR gene	VAF	Germline testing	History of LS-associated cancer		Cancer type	LS-associated
										Personal	Familial		
**1**	**M**	**65**	**F1**	**T**	**13.28**	**MSS**	***PMS2***	**0.4648**	**PMS2 (NM_000535 :c.241 G** > T p.E81*)	**No**	**Yes**	**lung cancer**	**unassociated**
**2**	**M**	**75**	**F1**	**T**	**4.83**	**MSS**	***MSH6***	**0.5298**	**Under investigation**	**No**	**Yes**	**colon cancer**	**associated**
**3**	**F**	**9**	**F1**	**T**	**191.65**	**MSS**	***PMS2***	**0.5069**	**PMS2 (NM_000535 :c.241 G** > T p.E81*) + Exon14 deficient (CMMRD)	**No**	**Yes**	**glioblastoma**	**associated**
**4**	**F**	**45**	**F1**	**T**	**23.96**	**MSI-H**	***MLH1***	**0.6391**	**MLH1 (NM_000249 : splice site 1731 G** > **A)**	**No**	**Yes**	**uterine cancer, intrahepatic cholangiocarcinoma**	**associated**
**5**	**F**	**58**	**F1**	**T**	**115.86**	**MSI-H**	***PMS2***	**0.4699**	**PMS2 (NM_000535 : splice site 1144** + **1 G** > **A)**	**No**	**Yes**	**uterine cancer**	**associated**
**6**	**M**	**74**	**NCC**	**P**	**0**	**MSI-H**	***MSH2***	**0.494**	**MSH2 c.211+1 G** > **C**	**Yes**	**Yes**	**pancreatic cancer**	**associated**
**7**	**M**	**72**	**F1**	**T**	**48.27**	**MSI-H**	***MSH2***	**0.465**	**MSH2 c.1255delC**	**No**	**Yes**	**small intestine cancer**	**associated**
**8**	**M**	**40**	**F1**	**T**	**33**	**MSI-H**	***MSH2***	**0.61**	**MSH2 c.211+1 G** > **C**	**No**	**Yes**	**colon cancer**	**associated**
**9**	**M**	**56**	**NCC**	**P**	**106**	**MSI-H**	***MSH2***	**0.47**	**MSH2 c.1216 C** > **T**	**No**	**Yes**	**colon cancer**	**associated**
**10**	**M**	**27**	**F1**	**T**	**177.78**	**MSI-H**	***MSH6***	**0.4872**	**Negative**	**No**	**Yes**	**colon cancer**	**associated**
**11**	**F**	**58**	**F1**	**T**	**3.62**	**MSS**	***PMS2***	**0.2405**	**Negative**	**No**	**Yes**	**uterine cancer**	**associated**
**12**	**F**	**60**	**F1**	**T**	**42.87**	**MSI-H**	***MSH6***	**0.3108**	**Negative**	**No**	**Yes**	**ovarian cancer**	**associated**
**13**	**M**	**70**	**F1**	**T**	**28.96**	**MSI-H**	***MSH2***	**0.7**	**Negative**	**No**	**Yes**	**prostate cancer**	**associated**
**14**	**M**	**36**	**F1**	**T**	**113.48**	**MSS**	***PMS2***	**0.7108**	**Declined**	**No**	**Yes**	**anaplastic astrocytoma**	**associated**
**15**	**F**	**45**	**F1**	**T**	**11.35**	**MSI-H**	***PMS2***	**0.4878**	**Declined**	**No**	**Yes**	**cervical cancer**	**unassociated**
**16**	**M**	**81**	**F1**	**T**	**7.24**	**MSS**	***PMS2***	**0.4636**	**Declined**	**No**	**No**	**lung cancer**	**unassociated**
**17**	**F**	**76**	**F1**	**T**	**5.04**	**unknown**	***MLH1***	**NA**	**Declined**	**No**	**Yes**	**myxofibrosarcoma**	**unassociated**
**18**	**F**	**64**	**F1LQ**	**T**	**1.26**	**MSS**	***MLH1***	**0.4753**	**Declined**	**No**	**Yes**	**intrahepatic cholangiocarcinoma**	**associated**
**19**	**M**	**59**	**F1**	**T**	**33.79**	**MSI-H**	***MLH1***	**0.5997**	**Declined**	**No**	**Yes**	**small intestine cancer**	**associated**
**20**	**M**	**64**	**F1**	**T**	**35**	**MSI-H**	***MLH1***	**0.96**	**Declined**	**No**	**Yes**	**lung cancer**	**unassociated**
**21**	**M**	**74**	**F1**	**T**	**25.34**	**MSI-H**	***MSH2***	**NA**	**Dead**	**No**	**No**	**prostate cancer**	**associated**

*T* tumor tissues only, *P* pair of tumor and normal tissues, *TMB* tumor mutational burden, *VAF* variant allele frequency, *CMMRD* constitutional mismatch repair deficiency syndrome

### Case presentation

#### Case #1

**Fig. 2:**
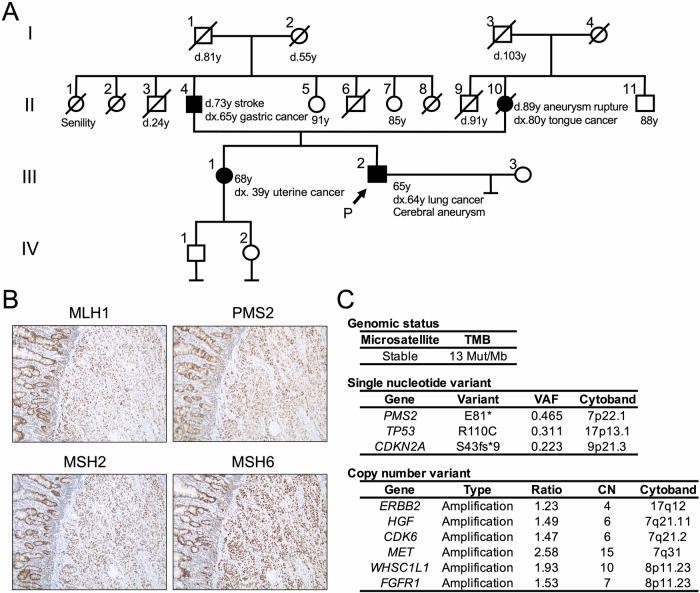
Patient 1. **A** A pedigree chart indicating the proband (arrow) and cancer-affected relatives (filled symbols). **B** Micrographs of immunohistochemistry for mismatch repair enzymes in the tumor. **C** Genetic alteration in comprehensive genomic profiling tests. Gene symbol and alteration in variants, and their variant allele frequency (VAF) are shown

A 65-year-old man with right lung adenocarcinoma underwent chemotherapy for multiple organ metastases (also referred to as case #1 in Table 1 and case #18 in Table 2). Although his sister had endometrial cancer, the patient did not have a personal history of LS-associated cancer (Fig. 2a). During chemotherapy, peritonitis developed due to bowel perforation, requiring emergency small bowel resection. Histopathological analysis revealed a small intestinal metastasis from the lung adenocarcinoma. MMR immunohistochemistry revealed no loss of MMR expression (Fig. 2b). Tissues from metastatic lesions were subjected to CGP. *PMS2* (p.E81*, VAF 0.465, NM_000535) was detected as a PGPV, whereas neither MSI-H nor high tumor mutational burden (TMB-high) was observed (Fig. 2c). Finally, germline testing was performed in order to confirm *PMS2* germline pathogenic variant.

**Table 2:** Summary of patients with Lynch syndrome who developed lung cancer

**Case**	**Age**	**Sex**	**Other cancer(age)**	**Family history(age)**	**NGS test**	**MSI-test**	**MMR-IHC**	**MMR gene**	**Genes with somatic mutations**	**Genetic testing of relatives**	**Author year**
**1**	**58**	**M**	**Colorectum (54, 58)**	**Father- leukemia(70)** **Brother- prostate(62)**	**―**	**―**	**dMMR**	**MSH2**			**,A. Canney, et al**. [31]
**2**	**64**	**M**	**Colorectum** (33, 53) **Bladder (51)** **Sebaceous neoplasms of skin** **(Muir-Torre syn.)**	**No**	**―**	**MSI-H**	**dMMR**	**MSH2**			**L. Nolan, et al. 2009**
**3**	**68**	**M**	**Colorectum** (**43, 63, 64)** **Lung(SCC)(63)** **Prostate(67)**	**Mother- uterus** **Brother- stomach**	**―**	**MSI-H**	**dMMR**	**MSH2**			**,Y. Kawashima, et al**. [32]
**4**	**74**	**F**	**No**	**No**	P*	**MSS**	**pMMR**	**PMS2**	**PTCH1**	**Yes**	**.S. Sun, et al**. [33]
**5**	**62**	**F**	**No**	**Mother- colon(40 s)**	**P**	**―**	**pMMR**	**MSH2**	**MAP2K2, GNAS**	**Yse**	**.S. Sun, et al**. [33]
**6**	**70-75**		**No**	**No**	**P**	**MSS**	**―**	**PMS2**	**GNAS,EGFR,MTOR,CUL3,CREBBP**	**No**	**.S. Sun, et al**. [33]
**7**	**55-60**		**No**	**Mother- colon**	**P**	**MSS**	**―**	**MSH6**	**MLL3,EGFR,TP53,RB1,NXF5,CBL**	**No**	**.S. Sun, et al**. [33]
**8**	**65-70**		**No**	**No**	**P**	**MSS**	**―**	**MSH6**	**IGF1R,TP53,ARID2,XRCC3,MET**	**No**	**.S. Sun, et al**. [33]
**9**	**75-80**		**No**	**Brother- stomach**	**P**	**MSS**	**―**	**MSH6**	**ALK,LRP1B,TP53,DNMT3A,HRAS,DDR2,NTM,TCF7L2,POLE**	**No**	**S. Sun, et al**. [33].
**10**	**50**	**M**	**Lung** **Stomach**	**Father- lung** **Sister- breast**	**P**	**―**	**―**	**MLH1** **MSH2**	**EGFR, TERT**	**Yes**	.**X.Chen et al**. [34]
**11**	**36**	**M**	**No**	**Sister- colorectum** (32) **Father- colorectum** (46, 54) **Paternal uncle- clorectum** (40) **Paternal grandmother-colorectum** (41)	**―**	**MSI-H**	**dMMR**	**MLH1**			**K.Masuzawa,et al. 2020**
**12**	**66**	**F**	**Endometorium**	**No description**	T**	**MSS**	**pMMR**	**MSH2**	**EGFR, TP53**		**.Hissong et al**. [35]
**13**	**74**	**F**	**Ovary** **Rectum**	**No**	**―**	**MSI-H**	**―**	**MSH6**			.**Maccaroni et al**. [36]
**14**	**76**	**M**	**No**	**Brother-stomach** **Sister-colon**	**P**	**―**	**pMMR**	**MSH6**	**ALK, LRP1B, TP53**	**Yes**	**,Y. Long, et al**. [37]
**15**	**62**	**M**	**No**	**No**	**P**	**MSI-H**	**dMMR**	**PMS2**	**KRAS, TP53**	**Yes**	**Q Han et al. 2022**
**16**	**70**	**F**	**Colon(51)** **Urothelium(63)** **Lung(67)**	**Father-colon(46)** **Uncle- colon(58)** **Brother- pancreas(52)** **Sister- colon(90),urothelium(70),HCC(80)**	**T**	**―**	**pMMR**	**MSH2**			**U.Majeed et al. 2023**
**17**	**62**	**F**	**Colon** **Tongue**	**Paternal grandmmother- colon** **Paternal aunt- colon** **Son- colon**(35)	**T**	**MSS**	**―**	**MLH1**	**EGFR**	**Yes**	**.A. Hodges et al**. [39]
**18** **(Our case)**	**65**	**M**	**No**	**Sister- endometrium**(39) **Father- stomach** **Mother- tongue**	**T**	**MSS**	**pMMR**	**PMS2**	**TP53, CDKN2A, ERBB2**	**No**	**Our case**

#### Case #2

**Fig. 3:**
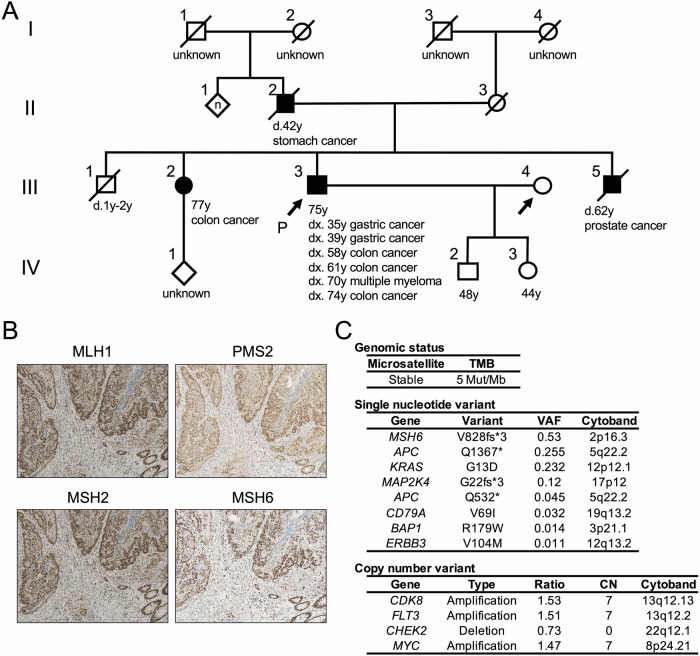
Patient 2. **A** A pedigree chart indicating the proband (arrow) and cancer-affected relatives (filled symbols). **B** Micrographs of immunohistochemistry for mismatch repair enzymes in the tumor. **C** Genetic alteration in comprehensive genomic profiling tests. Gene symbol and alteration in variants, and their variant allele frequency (VAF) are shown

A 75-year-old man who underwent colectomy for transverse colon cancer developed recurrent liver metastases(case #2 in Table 1). He had a history of multiple gastric and colorectal cancers, and three first-degree relatives had a history of LS-associated cancers (Fig. 3a). Although LS was strongly suspected based on rBG criteria, no loss of MMR expression was observed (Fig. 3b). CGP revealed PGPV in *MSH6* (V828fs*3, VAF 0.53, NM_000179; Fig. 3c). Similar to case #1, neither MSI-H nor TMB-high was observed in the CGP (Fig. 3c). Although a pathogenic variant of *APC* was detected, familial adenomatous polyposis coli was not suspected based on the absence of colonic polyposis or low VAF (Fig. 3c). Collectively, this was likely an LS, and a genetic confirmation test is under consideration.

### Analyzing the cancer genome atlas (TCGA)

**Fig. 4:**
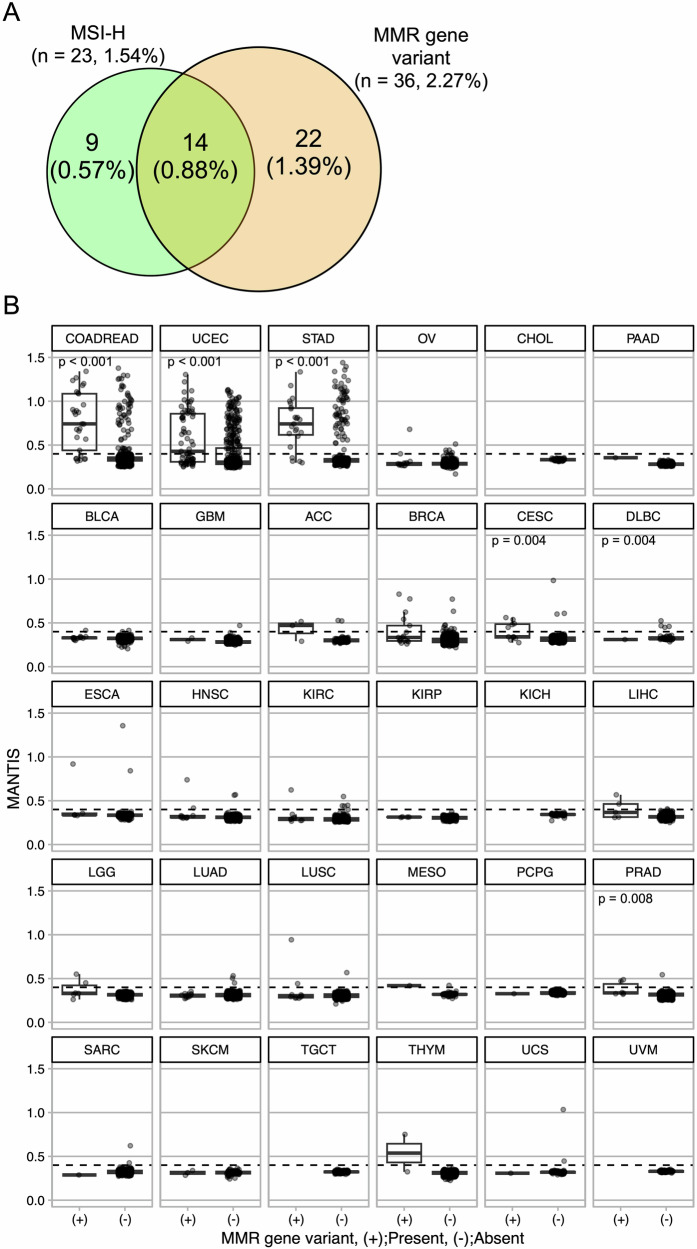
The association between microsatellite status and variant of mismatch repair gene (**A**) Venn diagram for microsatellite instability-high (MSI-H) and mismatch repair (MMR) gene variants in 1583 patients who underwent comprehensive genomic profiling tests (CGP) tests in Hiroshima University Hospital and the cooperative hospitals between January 2020 and April 2024. Percentages in the figures indicate the proportion in the 1583 patients. **B** Boxplots of MANTIS scores (y-axes) by the presence or absence of MMR gene variant (x-axes) in thirty types of cancers in The Cancer Genome Atlas (TCGA), Pancancer atlas dataset. Horizontal dashlines indicate 0.4 which is cut-off value of MANTIS score to determine microsatellite status (MSI-H ≥ 0.4 and MSS < 0.4) Wilcoxon’s rank-sum tests were performed and *p* values less than 0.05 were considered statistically significant. In the figure, the corresponding *p* values which were statistically significant are shown with individual boxplots

As in the two patients presented here, carriers of pathogenic variants of the MMR gene occasionally develop MSS. In fact, 22 of the 36 patients with MSS cancers and pathogenic variants of the MMR gene were identified in the CGP (Fig. 4A). Of these, eight were diagnosed with PGPV, and germline pathogenic variants of the MMR gene were confirmed in three of the four patients who underwent confirmatory genetic tests. This suggests that MSI-H is not always associated with pathogenic variants of MMR genes. Therefore, questions have been raised regarding the association between pathogenic variants of MMR genes and microsatellite status in various types of cancer. We first evaluated the microsatellite status in the TCGA Pancaner dataset by analyzing two MSI prediction scores, MANTIS and MSIsensor (Fig. 4B, Supplementary Fig. 1, and Supplementary Tables 3, 4). Overall, these two MSI prediction scores were significantly higher in colorectal (COADREAD), uterine (UCEC), and gastric (STAD) cancers than in other cancer types. Among these three cancer types, higher MSI prediction scores were observed in patients with pathogenic variants of MMR gene than in those without. We also found some MSI-H cancers without pathogenic variants of the MMR gene, presumably due to the epigenetic silencing of MMR expression. In contrast, the MSI prediction scores were found to be comparable in the presence and absence of pathogenic variants of the MMR gene in the other 24 cancer types (Fig. 4B, Supplementary Fig. 1, and Supplementary Tables 3, 4). Although the frequency of MSI-H cases was consistently higher in the presence of pathogenic variants of the MMR gene, the majority of microsatellite statuses retained MSS in most cases with pathogenic variants of the MMR gene, except in colorectal, uterine, and gastric cancers (Fig. 4B, Supplementary Fig. 1, and Supplementary Tables 3, 4).

## Discussion

Among the 1583 patients who underwent CGP tests, GPV and PGPV were detected in 2 and 19 patients, respectively. Among these 21 patients, seven had MSS cancer, 17 did not have colorectal cancer, and five had LS-unassociated cancers. Therefore, CGP is useful for screening patients with LS who cannot be diagnosed by conventional clinical screening such as the rBG criteria and MSI/MMR immunohistochemistry-based testing.

As observed in Case #1, it may be difficult to diagnose LS in patients with lung cancer. Lung cancer is extremely rare in patients with LS. In addition, this lung adenocarcinoma was neither MSI-high nor TMB-high, which made the diagnosis of LS difficult. Although we eventually determined the germline variant of *MSH2*, the *MSH2* pathogenic variant was found incidentally in the CGP test. There were only 18 cases of lung cancer in the LS when we reviewed previous literature and the current report (Table 2) [31–39]. In these cases, the diagnosis was difficult because only six patients showed MSI-H or deficient mismatch repair status. Among patients without a family history of LS-related cancer or MSI-H, only NGS-based tests provided the opportunity to be aware of LS, suggesting the usefulness of CGP for LS screening.

In our TCGA analysis, the MANTIS and MSI sensor scores were significantly higher in colorectal, uterine, and gastric cancers with pathogenic variants of the MMR gene than in those without MMR variants (Fig. 4B and Supplementary Fig. 1). MSI-H is frequently observed in patients without MMR variants, suggesting that sporadic MSI-H cancer is mediated by *MLH1* promoter methylation [3]. As MSI-H cancers are present in definite proportions of these types of cancers, screening by microsatellite status, followed by confirmatory genetic tests, is a reasonable strategy for LS diagnosis. Universal screening using MMR immunohistochemistry has been performed for colorectal and uterine cancers [12]. In addition to these cancers, universal screening for gastric cancers might be useful based on our findings in the TCGA analysis.

In contrast to colorectal, uterine, and gastric cancers, MSI-H was not frequently observed in other types of cancers with pathogenic variants of the MMR gene in TCGA dataset (Fig. 4 and Supplementary Fig. 1). This observation highlights the limitation of relying solely on MSI-based screening strategies to identify LS cases in non-colorectal cancers. Despite the absence of germline data, the TCGA analysis supports the need for broader genetic testing approaches beyond MSI status in such tumor types. Another report identified LS in 0.3% of patients with MSS tumors [40]. These findings would suggest there is a possibility of the existence of pathogenic variants of MMR gene in MSS cancers. In such cases, pathogenic variants of the MMR gene help us be aware of LS; however, it is unlikely to be a direct cause of cancer. In case #1, alterations in *TP53, CDKN2A, and ERBB2* appeared to directly cause lung cancer, according to the CGP results. In contrast, there might be fewer or no effects of *PMS2* germline pathogenic variants because the cancer itself did not exhibit MSI-H or MMR deficiency in CGP analysis and MMR immunohistochemistry.

Case #2 was more complicated. Based on the personal/familial history of cancer (Fig. 3A), hereditary cancer syndromes were suspected. In the CGP test, variants of *MSH6*, *APC*, and *CHEK2* were detected (Fig. 3C), all of which are listed in Kosugi’s recommendation list for secondary findings. The computed tumor purity, estimated by DNA sequencing of F1CDx, was 29.8%. Thus, if the variant was somatic, estimated VAF and copy number ratio would be ≤0.3 and ≥0.70, respectively. Both *APC or CHEK2* pathogenic variants were less likely to be germline variants, because either the VAF or copy number ratio was comparable to these estimates. In contrast, the VAF of 0.53 in *MSH6* was higher than the estimated VAF for the somatic variants. Based on this result, LS was highly suspected, even though the CGP test indicated MSS, and MMR immunohistochemistry revealed proficient MMR (Fig. 3B). We presumed *MSH6* germline pathogenic variant was unlikely to be a direct driver of this cancer and was discovered incidentally. In such cases, we might have missed the LS if we had focused only on MSS and proficient MMR using MMR immunohistochemistry. Although there is certainly a limitation of which the confirmatory genetic test has not been performed yet, case #2 gives us some insights for screening of LS by incidental MMR germline pathogenic variants.

This study has several limitations. First, we analyzed only a small number of patients from only twelve institutions. To complement this limitation, we have added a TCGA analysis. However, further large-scale studies are required to demonstrate the usefulness of CGP in screening for LS. Second, T-only CGP tests, which require confirmatory genetic tests when pathogenic variants of the MMR gene are detected, accounted for approximately 75% of our cohort, whereas confirmatory genetic tests were not performed in all patients because of patient choice or financial issues. Therefore, we could not confirm the actual detection rate of LS using CGP. Therefore, it is necessary to improve the performance of confirmatory genetic tests to obtain more conclusive results. Some ethical issues might have been raised because some confirmatory genetic tests were declined even after the disclosure of PGPVs. However, whether they underwent confirmatory genetic tests depended on the right to self-determination, and we provided genetic counseling to support the self-determination of secondary finding disclosure in all cases. The third limitation is the concordance of MSI testing between CGP-and conventional PCR-based testing. In Case #3, with a constitutional mismatch repair deficiency (Table 1), CGP showed MSS, whereas PCR-based MSI testing showed MSI-H, as reported previously [41]. It should be noted that the CGP results may differ among tests on certain occasions. Fourth, conventional clinical screening with MMR immunohistochemistry or MSI testing is usually performed for colorectal and uterine endometrial cancers at all stages. In contrast, CGP is provided for all advanced solid tumors for which the standard of care is almost complete. Although comparisons of sensitivity and specificity between CGP tests and conventional clinical screening are of great interest, we could not compare these two precisely because of differences in the nature of the cohort. Fifth, according to the National Cancer Institute Genomic Data Commons (NCI GDC), data on germline variants are not publicly disclosed, and we accessed only public datasets. Thus, all variants analyzed in TCGA dataset were somatic. Although critical, it is still useful to test whether an association exists between MMR pathogenic variants and microsatellite status in various types of cancers.

In conclusion, we have demonstrated the usefulness of CGP in LS screening using our case series and TCGA analysis. We should remember that only pathogenic variants of the MMR gene detected in the CGP provided the opportunity to be aware of the presence of LS, even though the patients did not have MSI-H cancers or personal/familial histories of LS-associated cancers.

## Supplementary information

Supplementary Tables 1-4

Supplementary Figure 1

Supplementary Figure legend

## Data Availability

The datasets used and/or analyzed during the current study are available from the corresponding author on reasonable request.
