# Supplementary material for: The usefulness of comprehensive genome profiling test in screening of Lynch syndrome independent of the conventional clinical screening or microsatellite instability tests
Source: J Hum Genet. 2025 May 8;70(8):385–93. doi: 10.1038/s10038-025-01345-x (PMC12289520; doi:10.1038/s10038-025-01345-x)
Supplement: Supplementary file 1 — Supplementary Tables 1-4 [file 10038_2025_1345_MOESM1_ESM.docx]

**Supplementary Table 1. Summary of comprehensive genomic profiling test.**

| Test | FoundationOne CDx | OncoGuide NCC Oncopanel System | GenMineTOP | FoundationOne Liquid CDx | Guardant360 CDx |
| --- | --- | --- | --- | --- | --- |
| Specimen | Tissue | Tissue | Tissue | Liquid | Liquid |
| DNA | T-only | T/N-paired | T/N-paired | T-only | T-only |
| Genes | 324 | 124 | 737 | 324 | 74 |
| RNA | No | No | 455 | No | No |
| GPV/PGPV | PGPV | GPV | GPV (59 genes) | PGPV | PGPV |
| Covered by insurance on | June 2019 | June 2019 | August 2023 | August 2021 | July 2023 |

T-only: CGP analyzing tumor tissue only, T/N-paired: CGP analyzing a matched pair tumor and normal tissues. GPV: germline pathogenic variant, PGPV: presumed germline pathogenic variant

**Supplementary Table 2. Summary of 1583 patients who underwent CGP.**

| Variables |  | All case | (%) |
| --- | --- | --- | --- |
| Age | Median (range) | 65 (4-92) |  |
| Sex | Male | 854 | 53.9 |
|  | Female | 724 | 45.7 |
| Cancer Type | Pancreas | 234 | 14.8 |
|  | Bowel/Colon | 187 | 11.8 |
|  | Biliary Tract | 172 | 10.9 |
|  | Lung | 152 | 9.6 |
|  | Prostate | 110 | 6.9 |
|  | Breast | 79 | 5.0 |
|  | Ovary | 71 | 4.5 |
|  | Soft Tissue | 62 | 3.9 |
|  | Uterus | 60 | 3.8 |
|  | CNS/Brain | 55 | 3.5 |
|  | Esophagus/Stomach | 55 | 3.5 |
|  | Head and Neck | 52 | 3.3 |
|  | Bladder/Urinary Tract | 48 | 3.0 |
|  | Liver | 30 | 1.9 |
|  | Kidney | 29 | 1.8 |
|  | Bone | 16 | 1.0 |
|  | Thyroid | 15 | 0.9 |
|  | Peritoneum | 14 | 0.9 |
|  | Adrenal Gland | 6 | 0.4 |
|  | Other | 136 | 8.6 |
| Type of CGP | FoundationOne CDx | 1196 | 75.6 |
|  | FoundationOne Liquid CDx | 164 | 10.4 |
|  | Guardant360 CDx | 94 | 5.9 |
|  | OncoGuide NCC Oncopanel System | 65 | 4.1 |
|  | GenMineTOP | 20 | 1.3 |

**Supplementary Table 3. The association between MMR gene variant and microsatellite status determined by MANTIS score**

|  | MMR gene variant (+) | | MMR gene variant (-) | |  |
| --- | --- | --- | --- | --- | --- |
| Cancer type | MSI-H | MSS | MSI-H | MSS | p-value |
| COADREAD | 22 | 7 | 64 | 437 | <0.001 |
| UCEC | 38 | 31 | 123 | 323 | <0.001 |
| STAD | 19 | 4 | 64 | 348 | <0.001 |
| ACC | 2 | 1 | 2 | 86 | <0.001 |
| BLCA | 1 | 12 | 2 | 395 | 0.0028 |
| BRCA | 5 | 12 | 11 | 990 | <0.001 |
| CESC | 5 | 6 | 3 | 277 | <0.001 |
| CHOL | 0 | 0 | 0 | 36 | N.A. |
| DLBC | 0 | 1 | 4 | 36 | N.A. |
| ESCA | 1 | 4 | 2 | 175 | 0.0011 |
| GBM | 0 | 2 | 1 | 386 | N.A. |
| HNSC | 2 | 9 | 2 | 490 | <0.001 |
| KICH | 0 | 0 | 0 | 65 | N.A. |
| KIRC | 1 | 8 | 4 | 305 | 0.0196 |
| KIRP | 0 | 4 | 0 | 272 | N.A. |
| LGG | 2 | 4 | 0 | 505 | <0.001 |
| LIHC | 2 | 3 | 1 | 358 | <0.001 |
| LUAD | 0 | 12 | 3 | 549 | N.A. |
| LUSC | 2 | 8 | 1 | 467 | <0.001 |
| MESO | 1 | 0 | 1 | 81 | <0.001 |
| OV | 2 | 11 | 5 | 402 | <0.001 |
| PAAD | 0 | 1 | 0 | 176 | N.A. |
| PCPG | 0 | 1 | 0 | 177 | N.A. |
| PRAD | 2 | 4 | 1 | 487 | <0.001 |
| SARC | 0 | 1 | 2 | 248 | N.A. |
| SKCM | 0 | 3 | 0 | 74 | N.A. |
| TGCT | 0 | 0 | 0 | 149 | N.A. |
| THYM | 1 | 1 | 0 | 607 | <0.001 |
| UCS | 0 | 1 | 2 | 54 | N.A. |
| UVM | 0 | 0 | 0 | 80 | N.A. |

Case number of MSI-H and MSS determined by MANTIS score in TCGA Pancancer dataset; MANTIS scores ≥0.4 and <0.4 are determined as MSI-H and MSS, respectively. Fisher exact tests were performed, and the corresponding p-value were indicated in the table. N.A.; not analyzed.

**Supplementary Table 4. The association between MMR gene variant and microsatellite status determined by MSIsensor score**

|  | MMR gene variant (+) | | | MMR gene variant (-) | | |  |
| --- | --- | --- | --- | --- | --- | --- | --- |
| Cancer type | MSI-H | MSI-int | MSS | MSI-H | MSI-int | MSS | p-value |
| COADREAD | 21 | 1 | 7 | 50 | 3 | 449 | <0.001 |
| UCEC | 29 | 11 | 29 | 99 | 26 | 323 | <0.001 |
| STAD | 15 | 4 | 4 | 54 | 9 | 350 | <0.001 |
| ACC | 0 | 2 | 1 | 0 | 2 | 86 | <0.001 |
| BLCA | 0 | 0 | 13 | 0 | 2 | 395 | N.A. |
| BRCA | 3 | 3 | 11 | 2 | 9 | 1030 | <0.001 |
| CESC | 1 | 3 | 7 | 3 | 0 | 277 | <0.001 |
| CHOL | 0 | 0 | 0 | 0 | 0 | 36 | N.A. |
| DLBC | 0 | 0 | 1 | 1 | 0 | 39 | N.A. |
| ESCA | 1 | 0 | 4 | 2 | 0 | 175 | 0.0011 |
| GBM | 0 | 0 | 2 | 0 | 2 | 385 | N.A. |
| HNSC | 1 | 1 | 9 | 0 | 4 | 488 | <0.001 |
| KICH | 0 | 0 | 0 | 1 | 7 | 57 | N.A. |
| KIRC | 1 | 0 | 9 | 4 | 4 | 387 | 0.0414 |
| KIRP | 0 | 0 | 4 | 0 | 0 | 272 | N.A. |
| LGG | 0 | 2 | 4 | 0 | 0 | 508 | <0.001 |
| LIHC | 1 | 1 | 3 | 0 | 0 | 359 | <0.001 |
| LUAD | 0 | 0 | 12 | 0 | 4 | 549 | N.A. |
| LUSC | 1 | 1 | 9 | 0 | 1 | 467 | <0.001 |
| MESO | 0 | 0 | 1 | 0 | 1 | 81 | N.A. |
| OV | 1 | 0 | 12 | 2 | 15 | 452 | 0.0038 |
| PAAD | 0 | 1 | 0 | 0 | 11 | 166 | 0.0002 |
| PCPG | 0 | 0 | 1 | 0 | 0 | 177 | N.A. |
| PRAD | 0 | 2 | 4 | 0 | 1 | 487 | <0.001 |
| SARC | 0 | 0 | 1 | 1 | 3 | 247 | N.A. |
| SKCM | 0 | 0 | 3 | 0 | 0 | 74 | N.A. |
| TGCT | 0 | 0 | 0 | 0 | 0 | 149 | N.A. |
| THYM | 1 | 0 | 1 | 0 | 0 | 607 | <0.001 |
| UCS | 0 | 0 | 1 | 1 | 0 | 55 | N.A. |
| UVM | 0 | 0 | 0 | 0 | 0 | 80 | N.A. |

Case number of MSI-H, MSI-intermediate (MSI-int) and MSS determined by MSIsensor scores in TCGA Pancancer dataset; MSIsensor scores ≥10, ≥3 and <10, and <3 are determined as MSI-H, MSI-int, and MSS, respectively. Chi-square tests were performed, and the corresponding p-value were indicated in the table. N.A.; not analyzed.
