# Supplementary figures and images for: The usefulness of comprehensive genome profiling test in screening of Lynch syndrome independent of the conventional clinical screening or microsatellite instability tests
Source: J Hum Genet. 2025 May 8;70(8):385–93. doi: 10.1038/s10038-025-01345-x (PMC12289520; doi:10.1038/s10038-025-01345-x)

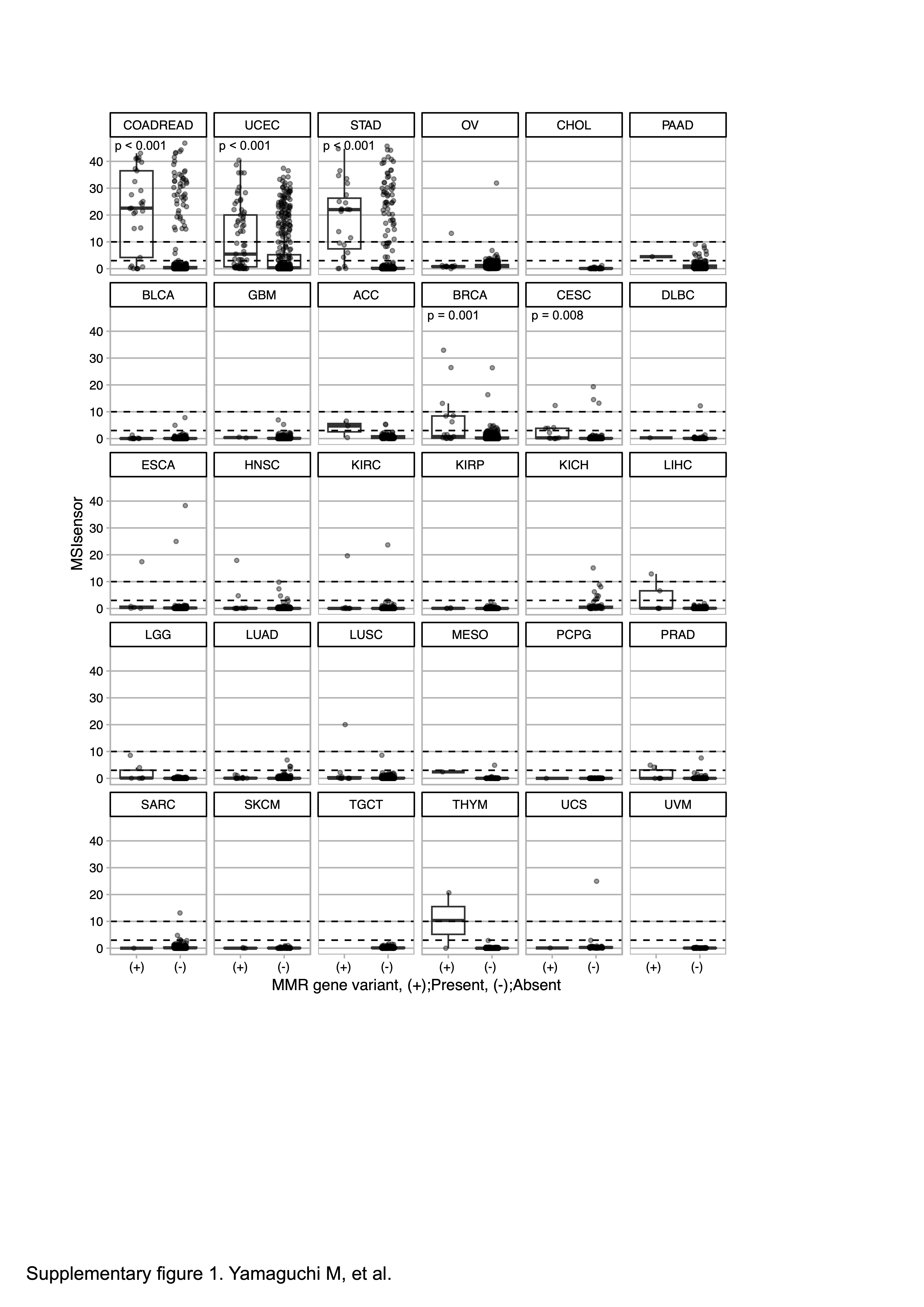

Supplement: Supplementary file 2 — Supplementary Figure 1 [file 10038_2025_1345_MOESM2_ESM.jpg]
