# Supplementary material for: The usefulness of comprehensive genome profiling test in screening of Lynch syndrome independent of the conventional clinical screening or microsatellite instability tests
Source: J Hum Genet. 2025 May 8;70(8):385–93. doi: 10.1038/s10038-025-01345-x (PMC12289520; doi:10.1038/s10038-025-01345-x)
Supplement: Supplementary file 3 — Supplementary Figure legend [file 10038_2025_1345_MOESM3_ESM.docx]

**Supplementary figure 1 (related to Figure 4B)**

Boxplots of MSIsensor scores by the presence or absence of MMR gene variant in thirty types of cancers in The Cancer Genome Atlas (TCGA), Pancancer atlas dataset. Lower and upper horizontal dashlines indicate 3 and 10 which are cut-off values of MSIsensor scores to determine microsatellite status ( MSI-H ≥10, MSI-indermediate ≥3 and <10, and MSS <3) Wilcoxon’s rank-sum tests were performed and p-values less than 0.05 were considered statistically significant. In the figure, the corresponding p-values which were statistically significant are shown with individual boxplots.
